# Hopeful News for Physicians Who Involved in the Treatment of Critical Aluminum Phosphide (Rice Pill) Poisoning Patients

**DOI:** 10.1155/2022/2418341

**Published:** 2022-10-20

**Authors:** Fatemeh Vafapour, Moslem Sedaghattalab

**Affiliations:** Department of Internal Medicine, Yasuj University of Medical Sciences, Yasuj, Iran

## Abstract

**Introduction:**

Aluminum phosphide (rice tablet) was first introduced as a pesticide in India. Rice tablets are commonly used in Iran due to their high efficacy against rodents and insects, low cost, and availability. Aluminum phosphide is a lethal poison without antidote and causes cardiocirculatory collapse and has negative inotropic cardiac effect. Human and animal studies showed that high dose insulin had positive cardiac inotropic effects. GIK (glucose, insulin, and potassium) assists heart uptake of carbohydrates that are the major fuel substrate of the myocard muscle under stressed conditions and leading to correction of acidosis, increased myocardial contractility, and peripheral vascular resistance. *Case Presentation*. In this manuscript, a young woman with aluminum phosphide poisoning was described to have presented with hypotension, hypoxemia, and severe metabolic acidosis. In contrast to our previous experiences that approximately all rice tablet poisoning patients with shock were dead despite full conservative treatment, this patient miraculously was saved with high dose intravenous regular insulin infusion and was discharged from the hospital with good condition and without any complications.

**Conclusion:**

Rice tablet poisoning has high fatality rate, and to date, no antidote is available. GIK is suggested as a potential life saving treatment for critical rice tablet poisoning patients with symptoms and signs of shock.

## 1. Introduction

Aluminum phosphide was first introduced as a pesticide in India. Rice pills are commonly used in Iran due to their high efficacy against rodents and insects, low cost, and availability. Aluminum phosphide poisoning has dramatically increased within the recent years due to its ingestion as a suicide drug. Rice tablet is a lethal poison without antidote and causes cardiocirculatory collapse and has negative inotropic cardiac effect [1].

Human and animal studies showed that high dose insulin had positive cardiac inotropic effects [2–4]. GIK assists heart uptake of carbohydrates that are the major fuel substrate of the myocard muscle under stressed conditions [5], and leading to correction of acidosis, increased myocardial contractility, and peripheral vascular resistance. High dose insulin was first introduced as a possible treatment of rice pill poisoning in 2008, although this treatment was has not proved in high power clinical randomized trials [1].

In this manuscript a young woman with aluminum phosphide poisoning was described to have presented with hypotension, hypoxemia, and severe metabolic acidosis. In contrast to our previous experiences that approximately all critical rice tablet poisoning patients with shock were dead despite full conservative treatment, this patient miraculously was saved with high dose intravenous regular insulin infusion and is discharged from the hospital with good condition.

## 2. Case Report

A 17-year-old young woman presented to emergency ward, Emam Sajad Hospital, Iran, with ingestion of one rice tablet 30 minutes before hospital admission, nausea, and vomiting. She also had a history of depression. Medication history was negative.

On examination, she was drowsy and Glasgow coma scale score (GCS) was 11, the body temperature, blood pressure, heart rate, oxygen saturation, and respiratory rate were 36.5°C, 80/60 mmHg, 130 beats/min, 80%, and 29 breaths/min, respectively.

Her extremities were cold, and all other examinations were normal. The patient was admitted in intensive care unit ward with hypotensive shock, severe hypoxemia; and intubation, mechanical ventilation, and other conservative treatment was initiated.

The platelet counts was 523000 (per *μ*l), hemoglobin 12 (g/dL), and the white blood cell counts 12000 (mm^3^). The alanine aminotransferase was 25 (IU/L), aspartate aminotransferase 70 (IU/L), albumin 3.4 (g/dL), alkaline phosphatase 99 (IU/L), total bilirubin 4.8 (mg/dL), conjugated bilirubin 0.3 (mg/dL), pH (potential of hydrogen) 7.12, CO_2_ (Carbon dioxide) 27 (mm Hg), HCO_3_ (bicarbonate) 10 (mEq/L), LDH (lactate dehydrogenase) 389 (U/ml), sodium 140 (mEq/L), potassium 3.7 (mEq/L), BUN (blood urea nitrogen) 28 (mg/dL), creatinine 0.4 (mg/dL), magnesium 2.3 (mg/dl), and blood sugar 123 (mg/dL). Other laboratory tests, such as troponin, partial thromboplastin time, prothrombin time, and blood culture were normal.

A CT (computerized tomography) scan of chest revealed mild pleural effusion. ECG (Electrocardiogram) revealed sinus tachycardia with mild and nonsignificant ST depression in inferior and lateral leads.

Treatment with sodium bicarbonate (to correct patient metabolic acidosis and to maintain serum bicarbonate above 20 mEq/L), hydrocortisone (100 mg doses at 8-hour intervals), high dose intravenous regular insulin (50 IU/hr, calculated by the formula 1 IU/kg/hr, based on previous studies (1)), potassium (maintain the serum potassium between 3.5-4.5 mEq/l), normal saline as maintenance serum therapy and loading dose for correction of hypotension, hypertonic dextrose water 50% (1 cc/kg/hr to maintain serum glucose between 100-180 mg/dL), intravenous pantoprazole for stress ulcer prophylaxis, subcutaneous enoxaparin (40 mg daily) for deep vein thrombosis prophylaxis and mechanical ventilation was initiated and the patient's symptoms and signs of hypoxemia and shock resolved completely and regular insulin was tapered after resolution of hypotension and acidosis and patient was transferred from intensive care unit ward after 4 days with good condition to general ward and was discharged from hospital without any complications.

## 3. Discussion

Aluminum phosphide tablets are a widely available and highly toxic without antidote. When these tablets come in contact with water, they quickly dissolve and emit phosphine gas. After ingestion of rice pill, phosphine gas is absorbed through the mucosa, causing nausea, vomiting, dyspnea, cardiac failure, refractory hypotension, and severe metabolic acidosis [6–9].

Mechanism of rice tablet toxicity includes cytochrome C oxidase inhibition by phosphine gas, oxidative injury, and lipid peroxidation that cause cellular injury. [6, 8, 9].

The most common cause of mortality in rice tablet poisoning is severe hypotension. It is commonly refractory to vasoactive agents such as norepinephrine and massive hydration with isotonic fluids such as normal saline. Other major causes of mortality in these patients are cardiogenic shock and severe metabolic acidosis, which is likely caused by cytochrome C oxidase inhibition and generalized tissue hypoperfusion. [10]

In circulatory shock, the metabolism or cellular uptake of glucose is impairing in the myocard muscle cells, resulting in a metabolic starvation, which additionally, worsens an already present toxin-induced cardiac depression. The proposed mechanism of hemodynamic benefit of high intravenous insulin and glucose is that it maximizes heart glucose uptake, enhance energy production from carbohydrates, and restitute calcium flux. The use of infusion in high dose insulin and dextrose in rice tablet poisoning was suggested after its beneficial positive inotropic heart effect in patients with severe beta-blockers and calcium channel blockers poisoning [5, 11–13].

It was determined in the study by Hassanian-Moghaddam et al. that administering high dose insulin (regular insulin 1 IU/kg/h-3 IU/kg/h followed by 0.2 to 0.5 U/kg/h with glucose and potassium) had significant effect on the decrease death in aluminum phosphide poisoning patients. Although their study has some limitations such as nonblinded randomization, low sample size (44 patients in intervention group), and dose of vasoactive support and other clinically relevant parameters were not investigated [1].

In a pilot study by Pannu et al. that administering GIK (insulin regular 0.1-0.5 IU/kg/h with glucose and potassium), they found that survival rate was significantly higher in intravenous insulin group, although their study was unblinded and had small sample size (30 patients in GIK group) and power [11].

In this manuscript a young woman with rice tablet poisoning was described to have presented with hypoxemia, severe metabolic acidosis, and hypotension. She had signs of severe shock and hypotension due to negative inotropic effect of phosphine and acidosis on heart. And her hypoxemia and metabolic acidosis were due to the inhibition of cytochrome C oxidase by phosphine and the formation of highly reactive free radicals.

In contrast to our previous experiences that approximately all critical rice tablets poisoning patients with hypoxemia and hypotension were dead, this patient at the height of disappointment was saved with high dose intravenous regular insulin and hypertonic dextrose water. Treatment with GIK, hydrocortisone, bicarbonate, mechanical ventilation, and other conservative treatment were initiated and the patient's signs of shock resolved completely and the patient was discharged from intensive care unit with good condition and without any complications.

## 4. Conclusion

Rice tablet poisoning has high fatality rate and to date, no antidote is available. In this manuscript a young woman with aluminum phosphide poisoning was described to have presented with hypoxemia, hypotension, and severe metabolic acidosis and successfully treated with high dose intravenous regular insulin and hypertonic dextrose, and discharged from intensive care unit with good condition. As a result, GIK is suggested as a potential life saving treatment for critical rice tablet poisoning patient with symptoms and signs of shock.
